# Controlling the Thermoelectric Performance of Doped Naphthobisthiadiazole‐Based Donor–Acceptor Conjugated Polymers through Backbone Engineering

**DOI:** 10.1002/advs.202410046

**Published:** 2024-11-03

**Authors:** Jian‐Fa Ding, Kodai Yamanaka, Shao‐Huan Hong, Guan‐Lin Chen, Wei‐Ni Wu, Jhih‐Min Lin, Shih‐Huang Tung, Itaru Osaka, Cheng‐Liang Liu

**Affiliations:** ^1^ Department of Materials Science and Engineering National Taiwan University Taipei 10617 Taiwan; ^2^ Graduate School of Advanced Science and Engineering Hiroshima University Higashi‐Hiroshima 739‐8527 Japan; ^3^ Center for Condensed Matter Sciences National Taiwan University Taipei 10617 Taiwan; ^4^ National Synchrotron Radiation Research Center Hsinchu 30076 Taiwan; ^5^ Institute of Polymer Science and Engineering National Taiwan University Taipei 10617 Taiwan; ^6^ Advanced Research Center for Green Materials Science and Technology National Taiwan University Taipei 10617 Taiwan

**Keywords:** backbone engineering, donor–acceptor, doped conjugated polymer, naphthobisthiadiazole, organic thermoelectric

## Abstract

This study investigates backbone engineering and evaluates the thermoelectric properties of FeCl_3_‐doped naphthobisthiadiazole (NTz)‐based donor–acceptor (D‐A) conjugated polymer films. The NTz acceptor unit is coupled with three distinct donor units, namely dialkylated terthiophene (3T), dialkylated quaterthiophene (4T), and dialkylated bisthienyl thienothiophene (2T‐TT) to yield copolymers designated as **PNTz3T**, **PNTz4T**, and **PNTzTT**. The difference in donor units leads to diverse molecule stacking and electronic properties, which can be systematically discovered via the three polymers. The linear structure of **PNTz4T** enables an orderly arrangement of side chains, thereby promoting dopant intercalation for enhanced carrier concentration. Additionally, this linear structure leads to an edge‐on stacking mode, thereby improving the in‐plane carrier mobility. As a result, the doped **PNTz4T** exhibits the highest electrical conductivity (*σ*) of 88.3 S cm^−1^ along with a Seebeck coefficient (*S*) of 62.2 µV K^−1^, thereby achieving the highest power factor (*PF*) of 34.2 µW m^−1^ K^−2^. These results highlight the relationship between the molecular design, microstructure, and doping effects in manipulating the thermoelectric performance of doped NTz‐based D‐A polymers.

## Graphical abstract

A series of doped naphthobisthiadiazole (NTz)‐based donor–acceptor conjugated copolymers with various donor units are explored for thermoelectric applications. Among them, the copolymer PNTz4T, featuring quaterthiophene (4T) as donor, exhibits the highest thermoelectric performance with a power factor of 34.2 µW m^−1^ K^−2^, due to its enhanced dopant interaction.

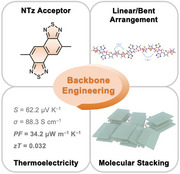

## 1 Introduction

Thermoelectric materials have recently gained significant attention for their ability to convert heat energy into electrical energy, making them a promising technology for effectively utilizing waste heat.^[^ ^1^, ^2^, ^3^, ^4^, ^5^, ^6^, ^7^, ^8^, ^9^, ^10^, ^11^, ^12^, ^13^, ^14^, ^15^, ^16^, ^17^, ^18^, ^19^, ^20^, ^21^, ^22^, ^23^, ^24^ ^]^ The efficiency of thermoelectric materials is commonly assessed using the dimensionless figure of merit (*zT*), which is defined by Equation (1):

(1) $$\;zT=S^{2}\sigma T\kappa^{-1}$$

where *σ* is the electrical conductivity, *S* is the Seebeck coefficient, *T* is the absolute temperature, and *κ* is the thermal conductivity. For organic‐based thermoelectric materials, the power factor (*PF*) is frequently employed as metric of performance, and is defined by Equation (2):

(2) $$\;PF=S^{2}\sigma$$

A primary challenge in maximizing the thermoelectric potential of polymer materials lies in augmenting their inherently low *σ* values, which consequently impacts their *PF* values. To address this issue, numerous studies have focused on doping conjugated polymers with various dopants to enhance the charge carrier concentration, thereby improving the electrical conductivity and overall thermoelectric performance.

Over the past decade, significant progress has been made in the development of donor–acceptor (D‐A) conjugated copolymers with enhanced thermoelectric performance.^[^ ^1^, ^2^, ^3^, ^11^, ^13^, ^14^, ^16^, ^22^, ^25^, ^26^, ^27^, ^28^, ^29^, ^30^, ^31^, ^32^, ^33^, ^34^, ^35^, ^36^, ^37^ ^]^ These polymers are characterized by narrow bandgaps and strong π–π stacking structures, and have proven advantageous for use in doped systems. For instance, Wang et al. investigated the thermoelectric performance of conjugated copolymers on polyfluorene and poly(2,7‐carbazole) derivatives, selecting fluorene and carbazole as the electron‐donor units and benzothiadiazole (BT) as the electron‐acceptor unit, while introducing the thiophene unit to tune the bandgap. Three polymers, **F8BT**, **F8TBT**, and **C8TBT**, were synthesized. The introduction of thiophene units in the **F8TBT** and **C8TBT** polymer backbones both reduced the bandgap and decreased the steric hindrance of the polymer chains, thereby promoting the formation of a coplanar structure and enhancing the electrical conductivity compared to **F8BT**. This resulted in optimal PF values of 1.81 and 13.11 µW m^−1^ K^−2^ for the **F8TBT** and **C8TBT**, respectively, which were approximately four times and twenty‐six times that of the **F8BT** (0.5 µW m^−1^ K^−2^). Additionally, Liu et al. investigated the thermoelectric properties of diketopyrrolopyrrole (DPP)‐based D‐A conjugated polymers by incorporating a 3,4‐ethylenedioxythiophene (EDOT) building block. They examined two polymers: **PDPP‐5T** and **PDPP4T‐EDOT** (where one thiophene unit was substituted with EDOT). The FeCl_3_‐doped **PDPP‐4T‐EDOT** exhibited an optimal *PF* of 298.2 µW m^−1^ K^−2^, while the doped **PDPP‐5T** film had a maximum *PF* of 11.1 µW m^−1^ K^−2^. The enhanced performance of the **PDPP‐4T‐EDOT** was attributed to the planar EDOT, which facilitates charge carrier transport after doping. To compare the thermoelectric performances of the conjugated D‐A polymers with thiophene or thieno[3,2‐b]thiophene donor units, Tam et al. employed benzo[1,2‐c;4,5‐c′]bisthiadiazole (BBT) as an acceptor and synthesized two polymers: **pBBT‐2T‐2T** and **pBBT‐2T‐TT**. They found that the triplet bipolaron was more stable in **pBBT‐2T‐TT** than **pBBT‐2T‐2T**. This enhanced the spin entropy, thereby leading to a larger Seebeck coefficient of 65.4 µV K^−1^ for the **pBBT‐2T‐TT**, compared to 46.6 µV K^−1^ for the **pBBT‐2T‐2T**. This led to *PF* values of 65.2 and 43.5 µW m^−1^ K^−2^ for the **pBBT‐2T‐TT** and **pBBT‐2T‐2T**, respectively. Similarly, the present research group investigated the thermoelectric performance of a series of doped BT‐based D‐A conjugated copolymers, where BT was selected as the acceptor and thiophene as the donor. The introduction of fluorene or side chains of various lengths on the thiophene resulted in polymers designated **PC16BTF**, **PC12BTH**, **PC16BTH**, and **PC20BTH**. Among these, the doped **PC16BTH** exhibited the highest *PF* of 22.4 µW m^−1^ K^−2^ due to its moderate side‐chain length and relatively high doping efficiency. Table S1 (Supporting Information) summarizes the recently published p‐type thermoelectric performance data for various doped D‐A conjugated polymers. However, further clarification of the relationship between the molecular structure and thermoelectric properties of the doped conjugated polymers is needed. Therefore, an investigation into the molecular engineering of the host conjugated polymer is required. Additionally, a suitable conjugated D‐A polymer for doping needs to be identified in order to optimize the thermoelectric performance.

**Figure 1:**
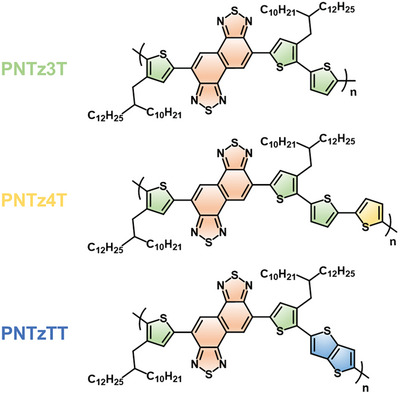
Chemical structures of NTz‐based D‐A conjugated polymers for doped polymer thermoelectric applications.

The naphtho[1,2‐c:5,6‐c′]bis[1,2,5]thiadiazole (NTz) unit provides both high planarity and extended π‐conjugation compared to the BT unit, thereby promoting enhanced charge mobility. Previous research has shown that NTz‐based conjugated polymers are well‐suited for organic solar cells (OSCs) and organic field‐effect transistors (OFETs). In Figure S1 (Supporting Information), we have organized the molecular structures and their performance. Notably, the field‐effect mobility of PNTz4T can exceed 0.5 cm^2^ V⁻¹ s⁻¹, and when oF2 is combined with fullerene and non‐fullerene acceptors, the power conversion efficiencies (PCEs) can reach up to 8.4% and 13.9%, respectively^[^ ^38^, ^39^, ^40^, ^41^, ^42^, ^43^ ^]^ In particular, NTz is recognized as a strong acceptor, and its incorporation into the conjugated polymer main chain alongside a donor unit is anticipated to decrease the energy level of the highest occupied molecular orbital (HOMO), which benefits the Seebeck coefficient. Building upon these findings, the present study explores the potential of doped NTz‐based D‐A conjugated polymers for thermoelectric applications. Here, polymers featuring NTz moieties polymerized with 3,3′“‐bis(2‐decyltetradecyl)‐2,2′:5′,2′”‐terthiophene (3T), 3,3′“”‐bis(2‐decyltetradecyl)‐2,2′:5′,2′“:5′”,2′“”‐quaterthiophene (4T), and 2,5‐bis(3‐(2‐decyltetradecyl)thiophen‐2‐yl)thieno[3,2‐*b*]thiophene (2T‐TT), designated as **PNTz3T**, **PNTz4T**, and **PNTzTT** respectively, are designed and synthesized. The chemical structures of the studied NTz‐based D‐A conjugated polymers are shown in **Figure** **1**. The results indicate that the conjugated backbone significantly impacts the molecular linearity and, hence, the molecular stacking. This, in turn, influences the position of dopant intercalation, which is crucial for the carrier concentration and mobility and, hence, the electrical conductivity. Additionally, the thermal conductivities of these three doped polymers are determined in order to evaluate their thermoelectric figures of merit (*zT* values). This work provides valuable insights into the backbone engineering of doped D‐A conjugated polymers for thermoelectric applications.

## 2 Results and Discussion

### 2.1 Synthesis and Characterization

The **PNTz3T**, **PNTz4T**, and **PNTzTT** were synthesized via copolymerization of the NTz‐monomer with distannylated monomers such as 2,5‐bis(trimethylstannyl)thiophene, 5,5′‐bis(trimethylstannyl)‐2,2′‐bithiophene,^[^ ^44^ ^]^ and 2,5‐bis(trimethylstannyl)thieno[3,2‐*b*]thiophene, respectively (Scheme S1, Supporting Information). The chemical structure of three polymers was characterized by ^1^H NMR spectra (Figure S2, Supporting Information). The molecular weights of the polymers were evaluated by high‐temperature gel permeation chromatography (GPC). The number‐average and weight‐average molecular weights (*M*_n_ and *M*_w_) of the **PNTz3T** were 33200 and 68900, respectively, and the dispersity (*Ð*) was 2.1 (**Table** **1**). The *M*_n_ and *M*_w_ of the **PNTz4T** were 25200 and 47600, with a *Ð* of 1.9, and those of the **PNTzTT** were 25600 and 51000 with a *Ð* of 2.0 (Table 1). Thermogravimetric analysis (TGA) revealed that the 5% weight loss temperatures (*T*_d_) of the **PNTz3T**, **PNTz4T**, and **PNTzTT** were 394, 358, and 308 °C, respectively (Figure S3a, Supporting Information). In addition, in the differential scanning calorimetry (DSC) measurements, the **PNTz3T** and **PNTz4T** each exhibited a peak due to melting at ≈300 °C, whereas the **PNTzTT** did not show any peaks in the range of *T*_d_ (Figure S3b, Supporting Information). These results suggest that the polymers are thermally stable.

**Table 1:** Molecular weight, thermal properties, optical properties, and electrochemical properties of synthesized NTz‐based D‐A conjugated polymers.

Polymer	*M_n_*	*M*_w_	*T_d_* [^o^C]	λ_max_ [nm]				*E*_g_^opt^ (eV)	*E*_HOMO_ ^a)^ [eV]	*E*_LUMO_ ^a)^ [eV]
				Solution		Thin film			
				π‐π transition	ICT	π‐π transition	ICT		
**PNTz3T**	33 200	68 900	394	428	659	445	740	1.53	−5.41	−2.87
**PNTz4T**	25 200	47 600	358	432	665	452	744	1.50	−5.39	−3.12
**PNTzTT**	25 600	51 000	308	438	671	449	746	1.49	−5.35	−2.90

^a)^ *E*_HOMO_ and *E*_LUMO_ are determined from CV

**Figure 2:**
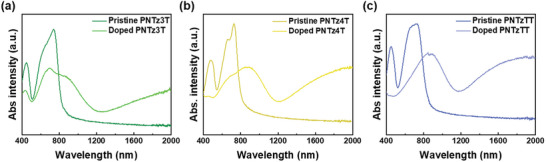
UV–vis–NIR absorption spectra of the pristine and doped thin film of a) PNTz3T, b) PNTz4T, and c) PNTzTT.

The Ultraviolet–visible–near infrared (UV–vis–NIR) spectra of the three pristine polymers as solutions in chloroform are presented in Figure S4 (Supporting Information), and the corresponding maximum absorption wavelengths (*λ*_max_) and optical energy gaps (*E*_g_^opt^) are summarized in Table 1. All of the spectra exhibit two characteristic absorption peaks typical of D‐A type polymers. The first of these appears at 428, 432, and 438 nm for the **PNTz3T**, **PNTz4T**, and **PNTzTT**, respectively, and is associated with localized π‐π transitions. The second absorption peak occurs at 659, 665, and 671 nm, respectively, and is attributed to intramolecular charge transfer (ICT) absorption.^[^ ^45^, ^46^, ^47^ ^]^ Notably, the **PNTzTT** exhibits a more pronounced red‐shift in both peaks compared to the others, which can be attributed to the more vital interaction and π‐conjugated system imparted by the thieno[3,2‐b]thiophene moieties. The UV‐Vis‐NIR spectra for the polymer thin films are presented in **Figure** **2**, where the peaks corresponding to localized π‐π transitions are observed at 445, 452, and 449 nm for the **PNTz3T**, **PNTz4T**, and **PNTzTT**, respectively. Meanwhile, the main peak for each conjugated polymer is that due to intermolecular charge transfer, which appears at 740, 744, and 746 nm, respectively. Thus, both peaks exhibit a red‐shift compared to the solution state in each polymer, which can be attributed to polymer aggregation and increased intramolecular interactions in the thin film state. Further, the **PNTz3T**, **PNTz4T**, and **PNTzTT** have *E*_g_^opt^ values of 1.53, 1.50, and 1.49 eV, respectively. Thus, the **PNTz4T** and **PNTzTT** exhibit more significant red‐shifts in their primary absorption peaks, along with slightly lower energy gaps compared to the **PNTz3T**. This is caused by the enhanced π‐conjugated system.

The energy levels of the three NTz‐based D‐A conjugated polymers are elucidated by the cyclic voltammetry (CV) curves of the thin films in Figure S5 (Supporting Information). The polymer thin films were cast from CB solution directly on the working electrode and the measurement was carried out with an Ag/Ag^+^ reference electrode in acetonitrile containing tetrabutylammonium hexafluorophosphate (0.1 m). The highest occupied orbital energy levels (*E*_HOMO_) and lowest unoccupied orbital energy levels (*E*_LUMO_) were calculated using the onset of the oxidation and reduction potential, respectively, which were calibrated by the the half‐wave potential of the ferrocene/ferrocenium redox couple measured under identical condition (see Figure S5, Supporting Information for redox potentials). The numerical results are summarized in Table 1. Thus, the **PNTz3T** has the lowest (most negative) *E*_HOMO_ of −5.41 eV, followed by −5.39 and −5.35 eV for the **PNTz4T** and **PNTzTT**, respectively. Thus, as the π‐conjugated system enlarges, the *E*_HOMO_ shifts toward a lower value. Meanwhile, the *E*_LUMO_ values are −3.17, −3.12, and −3.20 eV for the **PNTz3T**, **PNTz4T**, and **PNTzTT**, respectively.

### 2.2 Theoretical Calculations

**Figure 3:**
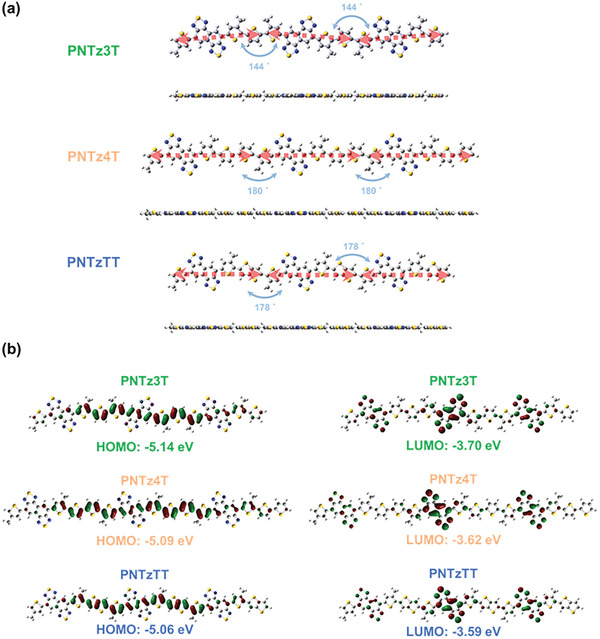
a) Top and side view molecular structures of PNTz3T, PNTz4T, and PNTzTT. b) DFT‐optimized molecular frontier orbitals of PNTz3T, PNTz4T, and PNTzTT.

Theoretical calculations using density functional theory (DFT) were conducted to optimize the molecular structure and energy states of the three polymers. The molecular simulations utilized three repeating units of each polymer, with the alkyl chains being replaced by methyl groups for computational simplicity. These simulations were performed using the B3LYP functional and the 6–31G basis set. The top and side views of the studied model compounds are shown in **Figure** **3**a. Here, it is evident that the three NTz‐based D‐A conjugated polymers exhibit high planarity, which is beneficial for charge carrier transfer. Further, while the **PNTz3T** model compound adopts a bent arrangement along its backbone, with an angle of 144° between extending directions of two adjacent repeating units, the **PNTz4T** exhibits a more linear arrangement. This difference influences the molecular stacking and potentially the thermoelectric properties, as discussed later. The calculated HOMO and LUMO energy levels are illustrated in Figure 3b. Similar electron distributions are observed in all three model compounds, such that the electron density of the LUMO is localized on the NTz units, while the electron density of the HOMO is delocalized over the polymer backbone and extends partially to the thiophene units. The *E*_HOMO_ obtained from the molecular simulation is located at −5.14, −5.09, and −5.06 eV for the **PNTz3T**, **PNTz4T**, and **PNTzTT**, respectively, which is consistent with the results obtained from the CV curves. The **PNTzTT** has the lowest *E*_HOMO_, thereby indicating that the **NTzTT** possesses a more robust π‐conjugated system than the other model compounds.

### 2.3 Spectroscopic Analysis of Doped Conjugated Polymers

For the doping procedure, the polymer films were immersed in a 3 mm FeCl_3_/acetonitrile solution for 30 s, resulting in color changes from dark green to blue. The UV–vis–NIR spectra of the three doped polymer thin films are presented in Figure 2. Thus, after FeCl_3_ doping, the primary peaks of the conjugated polymers are seen to be bleached, with polaron and bipolaron bands appearing in the regions of 800–1000 nm and beyond 1600 nm, respectively. This phenomenon suggests that the dopant causes charge transfer, leading to an increase in the number of charge carriers. While polarons typically improve electrical conductivity, bipolarons can also raise charge carrier concentration, but their effect on conductivity may be less positive compared to polarons. Notably, the **PNTz4T** exhibits higher polaron and bipolaron bands than the **PNTz3T**, which can be attributed to improved miscibility between the polymer and dopant upon introduction of additional thiophene units in the backbone. Similarly, the thieno[3,2‐b]thiophene moieties in the **PNTzTT** lead to increased molecular interactions compared to the **PNTz3T**, thus leading to higher polaron and bipolaron bands in the former.

**Figure 4:**
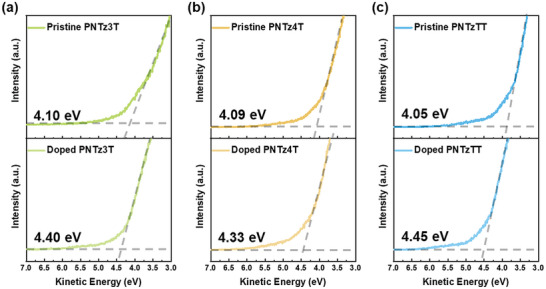
UPS spectra showing the kinetic energy region and the determined Fermi level of pristine and doped thin film of a) PNTz3T, b) PNTz4T, and c) PNTzTT.

The low kinetic energy regions of the pristine and doped conjugated polymers are determined, and their Fermi levels (*E*_F_) confirmed, by the ultraviolet photoelectron spectroscopy (UPS) results in **Figure** **4**. Thus, the pristine **PNTz3T**, **PNTz4T**, and **PNTzTT** exhibit Fermi levels of 4.10, 4.09, and 4.05 eV, respectively, where the smaller Fermi level of the **PNTzTT** is attributed to the enlarged π‐conjugated system provided by thieno[3,2‐b]thiophene. Upon doping, the Fermi levels of are shifted to higher values of 4.40, 4.33, and 4.45 eV, respectively. This shift in the Fermi level is characteristic of typical p‐type doping, thereby confirming the effectiveness of the doping process in altering the electronic properties of the polymers and facilitating an enhanced charge carrier density. We have compiled a schematic energy level diagram based on CV, theoretical calculations, and UPS experimental data, as shown in Figure S6 (Supporting Information).

**Figure 5:**
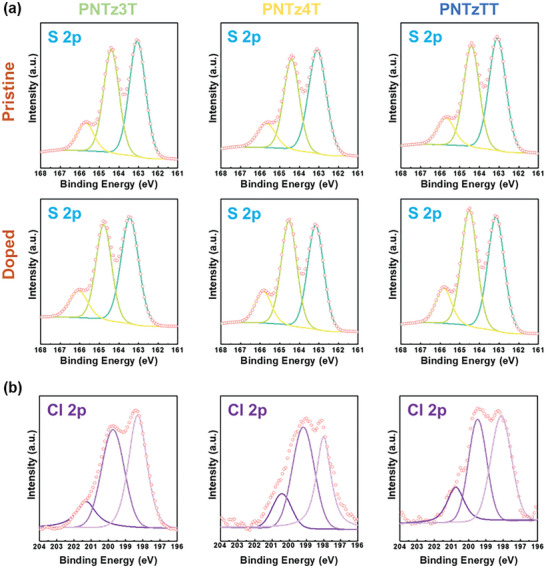
a) XPS spectra of the S 2p signal. b) XPS spectra of the Cl 2p signal for pristine and doped PNTz3T, PNTz4T, and PNTzTT.

The chemical shifts of the pristine and doped conjugated polymer thin films are compared by the X‐ray photoelectron spectroscopy (XPS) results in **Figure** **5**. This analysis focuses on the sulfur (S) and chlorine (Cl) signals to elucidate the chemical states and interactions of the dopant with the polymer backbone.^[^ ^48^, ^49^ ^]^ First, the chemical state of the conjugated polymer backbone is elucidated by the sulfur (S) 2p signal (Figure 5a). This is deconvoluted into three peaks due to oxidized sulfur (yellow line), S 2p_1/2_ (green line), and S 2p_3/2_ (bright green line) at binding energies of 165.7, 164.4, and 163.1 eV, respectively, for the pristine polymers. After doping, the S 2p_1/2_ and S 2p_3/2_ peaks are shifted to higher binding energies of 164.5 and 163.4 eV, respectively, for all three polymers. Notably, the intensity of the S 2p_1/2_ peak increases significantly for the doped **PNTz4T** and **PNTzTT** compared to their pristine forms, while the **PNTz3T** shows a less pronounced increase. This indicates that the **PNTz4T** and **PNTzTT** have stronger interactions with the dopant than does the **PNTz3T**. These dopant interactions are further elucidated by the Cl 2p signal in the binding energy range of 196–202 eV in Figure 5b. Upon dissolution in the solvent, FeCl_3_ dissociates into FeCl_4_^–^ and FeCl_2_^+^ ions. The FeCl_2_^+^ ions then undergo reduction to form FeCl_2_, and Cl^–^ anions. Due to coulombic interactions, the Cl^–^ anions coexist with the unreacted FeCl_4_^–^ anions within the polymer chains. Thus, the Cl signal is decomposed into three peaks at 200.7, 199.5, and 198.1 eV due to FeCl_2_^+^, FeCl_2_, and Cl^–^/FeCl_4_^–^, respectively. Based on these results, the doping efficiency (*η_d_*) is calculated using Equation (3)

(3) $$\eta_{d}=\frac{A^{-}}{A^{0}+A^{-}}$$

where *A* ^0^ is the area under the FeCl_2_^+^ peak and *A^–^* is the combined area under the Cl^–^ and FeCl_2_ peaks. Thus, the doping efficiencies of the **PNTz3T**, **PNTz4T**, and **PNTzTT** are found to be 0.90, 0.89, and 0.88, respectively. Although there is no significant difference in doping efficiency, the **PNTz4T** and **PNTzTT** exhibit more robust FeCl_2_ signals than the **PNTz3T**, thereby indicating a higher degree of FeCl_3_ reduction and more significant charge transfer, which is consistent with the UV‐Vis‐NIR spectroscopic results. Additionally, we have quantified the Fe dopant loading through XPS analysis. The Fe dopant constitutes a relatively small proportion of the overall thin film system for PNTz3T, PNTz4T, and PNTzTT, as presented in Tables S3–S5 (Supporting Information), respectively.

### 2.4 Morphological and Microstructural Analysis

**Figure 6:**
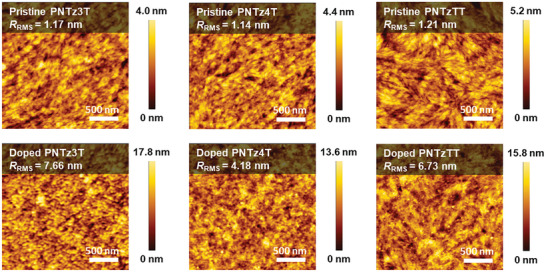
AFM height images and the root‐mean‐square surface roughness (*R*_RMS_) of pristine (upper) and doped (lower) polymer thin film.

The miscibility of the dopants within each conjugated polymer is reliably investigated by examining the atomic force microscopy (AFM) images in **Figure** **6**. Due to the planarity of the NTz moiety, all three polymers exhibit self‐aggregation on their surfaces.^[^ ^46^, ^47^, ^50^ ^]^ Notably, the surface of the pristine **PNTzTT** (Figure 6) displays a needle‐like structure, which can be attributed to the stronger intermolecular interactions facilitated by the thieno[3,2‐b]thiophene units, in agreement with the UV‐Vis‐NIR spectra. Consequently, the pristine **PNTzTT** exhibits a more significant root‐mean‐square surface roughness (*R*_RMS_) than the **PNTz3T** and **PNTz4T**, with *R*_RMS_ values of 1.21, 1.17, and 1.14 nm, respectively. Upon doping with FeCl_3_, dopant aggregation is revealed by bright dots in the AFM images (Figure 6). Moreover, the density of these bright dots inversely correlates with the doping miscibility. In the case of the **PNTz3T**, the dots are densely clustered, leading to a significant increase in *R*_RMS_ to 7.66 nm due to dopant aggregation. For the **PNTz4T**, the bright dots are more dispersed, resulting in the lowest *R*_RMS_ of 4.18 nm. Notably, the **PNTzTT** retains its needle‐like structure after doping, with finer and more uniformly distributed precipitates compared to the other polymers. Nevertheless, this polymer exhibits an intermediate *R*_RMS_ of 6.73 nm, which can be attributed to the inherent valleys and summits of its needle‐like structure.

**Figure 7:**
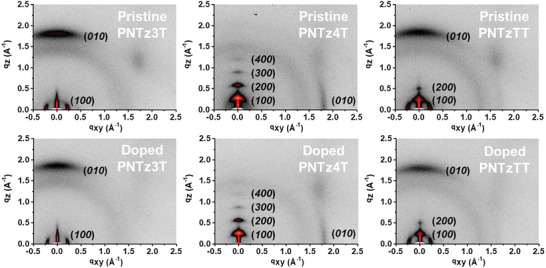
2D‐GIWAXS patterns of pristine (upper) and doped (lower) polymer thin films.

The microstructures of the three polymers are further elucidated by the 2D grazing‐incidence wide‐angle X‐ray scattering (GIWAXS) patterns in **Figure** **7**, along with the in‐plane and out‐of‐plane line cut patterns in Figure S7 (Supporting Information). Further, the molecular stacking behaviors are investigated by calculating the various crystallographic parameters of the pristine and doped conjugated polymers films, including the lamellar distance, π‐π distance, full width at half maximum (FWHM) of the (*100*) peak, and corresponding coherent length (*L*_c_), as summarized in **Table** **2**. For the pristine polymer films, the **PNTz3T** exhibits a (*100*) diffraction peak at 0.25 Å^−1^ on the *q_xy_* axis and a (*010*) diffraction peak at 1.84 Å^−1^ on the *q_z_* axis, and is characterized by a face‐on stacking mode. With the increased thiophene content in the **PNTz4T**, the orientation shifts to an edge‐on stacking mode, as indicated by the (*h00*) peaks on the *q_z_* axis and the (*010*) diffraction peak at 1.84 Å^−1^ on the *q_xy_* axis. This edge‐on orientation is beneficial for in‐plane charge carrier transport via hopping through the polymer backbone, which potentially enhances the carrier mobility and electrical conductivity. Meanwhile, the **PNTzTT** exhibits both (*h*00) diffraction peaks and a (*010*) diffraction peak at 1.90 Å^−1^ on the *q_z_* axis, along with a (*100*) diffraction peak at 0.26 Å^−1^ on the *q_xy_* axis, thereby indicating mixed edge‐on/face‐on orientations. Thus, the three polymers exhibit quite distinct stacking modes, which are further confirmed by the pole figures of the (*010*) peaks in Figure S8 (Supporting Information), where the (*010*) peak intensity is recorded as a function of the polar angle, and by the ratio between the face‐on and edge‐on portions, which are defined by the integrated intensities of the (*010*) reflections at χ = 0–45° and 45–90°, respectively. Thus, the **PNTz3T** demonstrates a relatively strong intensity at ≈90° and a high face‐on/edge‐on fraction of 2.41, thereby indicating predominantly face‐on stacking, while the **PNTzTT** exhibits a relatively low face‐on/edge‐on fraction of 1.18, representing a mixed orientation. Meanwhile, the **PNTz4T** exhibits a face‐on/edge‐on fraction of only 0.2, thereby indicating a predominantly edge‐on orientation. The differences between the stacking modes of the three polymers can be attributed to the degree of linearity observed in the molecular simulations. Previous work has shown that an ordered alignment of the side chains leads to an edge‐on orientation, where the alignment is influenced by the linearity of the conjugated backbone. A linear backbone allows the side chains to make close contact with the flat substrate in order to reduce the interfacial free energy in a similar chain conformation, thus leading to edge‐on stacking. By contrast, a bent backbone interrupts the alignment of side chains on the flat substrate, so that the backbone tends to lie down and adopt a face‐on contact rather than an edge‐on contact.^[^ ^51^ ^]^ Hence, the bent **PNTz3T** molecule exhibits face‐on stacking, while the **PNTz4T**, with the most linear backbone, exhibits edge‐on stacking, and the **PNTzTT** with its slightly bent shape exhibits both edge‐on and face‐on stacking.

**Table 2:** Summary of GIWAXS key features for pristine and doped polymers.

	Polymers	π ‐ π (010) spacing [Å]	Lamellar (100) spacing [Å]	FWHM (100) [Å^−1^]	*L_c_* (100) [Å]
PNTz3T	pristine	3.4	25.2	0.035	211.0
	doped	3.4	27.4	0.036	191.1
PNTz4T	pristine	3.4	22.1	0.028	349.7
	doped	3.5	22.3	0.033	269.5
PNTzTT	pristine	3.3	23.8	0.038	170.6
	doped	3.3	24.5	0.041	134.9

**Figure 8:**
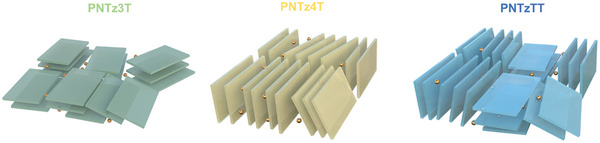
Illustration of the orientation of doped PNTz3T, PNTz4T, and PNTzTT.

As shown in Table 2, the pristine **PNTz3T** and **PNTzTT** exhibit greater lamellar distances of 25.2 Å and 23.8 Å, respectively, compared to 22.1 Å for the pristine **PNTz4T**, thereby indicating that a more orderly alignment of the side chains decreases the lamellar distance. Meanwhile, the π‐π distances of the **PNTz3T**, **PNTz4T**, and **PNTzTT** are 3.4, 3.4, and 3.3 Å, respectively. The differences in lamellar distance and π‐π spacing before and after doping suggest the preferred sites for dopant intercalation. As illustrated in **Figure** **8**, each polymer maintains the same orientation before and after doping. In the case of the **PNTz3T** and **PNTzTT**, the dopants preferentially intercalate in the side‐chain sites, thus leading to an increase in the lamellar spacing, which is likely to impede the effective charge transfer due to the lack of a conjugated system on the side chain. Specifically, the lamellar spacing of the **PNTz3T** increases by 2.2 Å upon doping, while the π‐π spacing remains unchanged. However, a lesser increase in lamellar spacing (by 0.7 Å) is observed for the **PNTzTT**, which is attributed to the face‐on stacking of this molecule and facilitates the diffusion of the dopant. By contrast, the lamellar spacing of the **PNTz4T** increases by 0.2 Å, and the π‐π spacing increases by 0.1 Å, thereby suggesting that the dopants prefer to intercalate themselves within the co‐facial polymer backbone, which is beneficial for efficient interaction between the dopant and the conjugated system.

The impact of the dopant on the microstructure is further elucidated by the *L*_c_ values of the three polymer thin films, which correspond to the sizes of the ordered stacking regions. Specifically, the *L*_c_ value of the **PNTz3T** decreases from 211 Å before doping to 191 Å after doping, while that of the **PNTz4T** decreases from 349 to 269 Å, and that of the **PNTzTT** decreases from 176 to 134 Å, thus implying that the dopant intercalation hinders the orderly stacking of the polymers. In both the pristine and doped samples, the **PNTz3T** and **PNTz4T** exhibit higher *L*_c_ values than the **PNTzTT**, thereby indicating a relatively less ordered stacking in the latter, which is possibly due to its mixed orientations in the thin films. This characteristic significantly impacts the charge transport capability.

### 2.5 Thermoelectric Properties

**Figure 9:**
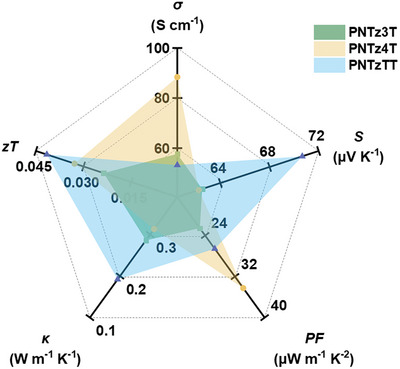
Chart of *σ*, *S*, *PF*,*κ*, and *zT* of doped PNTz3T, PNTz4T, and PNTzTT thin films.

The thermoelectric parameters of the doped conjugated polymer thin films, including the electrical conductivity, Seebeck coefficient, and power factor, as measured in the in‐plane dimension by using commercial equipment under a helium atmosphere, are presented in **Figure** **9** and Table S2 (Supporting Information). Thus, the doped **PNTz3T**, **PNTz4T**, and **PNTzTT** exhibit electrical conductivities of 57.7, 88.3, and 53.2 S cm^−1^, respectively. The electrical conductivity is typically influenced by both the carrier concentration and carrier mobility. Hence, the electrical conductivity is further elucidated by the Hall effect measurements in Table S4 (Supporting Information), indicating carrier mobilities of 2.7 × 10^−1^, 3.2 × 10^−1^, and 2.4 × 10^−1^ cm^2^ V^−1^ s^−1^, and carrier concentrations of 4.2 × 10^20^, 5.2 × 10^20^, and 4.0 × 10^20^ cm^−3^, for the doped **PNTz3T**, **PNTz4T**, and **PNTzTT**, respectively. The doped **PNTz4T** exhibits an outstanding electrical conductivity due to the high carrier mobility provided by the edge‐on stacking and enhanced carrier concentration arising from the enhanced interactions between the linear conjugated backbone and the dopant. By contrast, the doped **PNTz3T** exhibits a reduced carrier concentration due to less effective doping, as indicated by the GIWAXS results, which is consistent with the lower polaron and bipolaron levels evidenced by the UV‐vis‐NIR spectroscopy, thus leading to lower electrical conductivity than the doped **PNTz4T**. Meanwhile, the doped **PNTzTT** exhibits the lowest carrier mobility and carrier concentration due to its rougher surface and mixed mode of orientation, which impede the charge carrier transport. Therefore, the **PNTzTT** displays the lowest electrical conductivity. The electrical conductivity is not only related to carrier concentration but also to the Seebeck effect, as indicated by Equation (4):

(4) $$S\;=\;\frac{8\pi^{2}\;k_{B}^{2}\;T}{3eh^{2}}m\;{\left(\frac{\pi }{3n}\right)}^{\frac{2}{3}}$$

where *k*_B_ is Boltzmann's constant, *T* is the absolute temperature, *e* is the electron charge, *m* is the mass of the electron, and *n* is the charge‐carrier concentration. Due to this trade‐off relationship, the **PNTzTT**, with the lowest charge carrier concentration, has the highest Seebeck coefficient of 70.6 µV K^−1^, while the **PNTz3T** and **PNTz4T** have Seebeck coefficients of 62.5 and 62.2 µV K^−1^, respectively. Consequently, the *PF* values of the **PNTz3T**, **PNTz4T**, and **PNTzTT** are 22.5, 34.2, and 26.5 µW m^−1^ K^−2^, respectively. Thus, the **PNTz4T** has the highest *PF* due to the superior electrical conductivity facilitated by efficient in‐plane charge transport. Meanwhile, despite having the highest Seebeck coefficient, the **PNTzTT** ranks second in *PF*. Compared to previous work on benzothiadiazole (BT)‐based D‐A conjugated polymers, the NTz‐based copolymers, with their doubly BT‐fused rings, exhibit lower (more negative) *E*_HOMO_ values and higher electron affinities.^[^ ^39^, ^41^, ^43^, ^52^ ^]^ These effectively moderate the carrier concentration, which benefits the Seebeck coefficient more than the BT‐based counterparts.^[^ ^4^, ^16^, ^26^, ^27^, ^30^ ^]^ As a result, the NTz‐based D‐A conjugated polymers with their highly π‐extended structures achieve higher *PF* values than BT‐based D‐A conjugated copolymers such as **PC16BTH**, with a *PF* of 22.4 µW m^−1^ K^−2^.

The performances of the doped polymer thin films are comprehensively evaluated by the time‐domain thermoreflectance (TDTR) measurements of thermal conductivity (*κ*) and corresponding figure of merit (*zT*) values in Tables S2 and S3 (Supporting Information). Here, the pristine **PNTz3T**, **PNTz4T**, and **PNTzTT** thin films exhibit *κ* values of 0.24, 0.28, and 0.16 W m^−1^ K^−1^, respectively. In these undoped polymer thin films, a low carrier concentration results in a phonon‐dominated thermal conductivity, with the **PNTzTT** having the lowest value due to its mixed orientations, which disturb the phonon transport.^[^ ^15^, ^28^ ^]^ After doping, the thermal conductivity increases to 0.28, 0.31, and 0.19 W m^−1^ K^−1^ for the doped **PNTz3T**, **PNTz4T**, and **PNTzTT**, respectively. This increase is attributed to the enhanced carrier concentration from dopant transfer, which provides more carriers for efficient heat transport. Meanwhile, the **PNTzTT** exhibits the highest *zT* of 0.041, followed by 0.032 and 0.023 for the **PNTz4T** and **PNTz3T**, respectively. Thus, the lowest thermal conductivity of the **PNTzTT** contributes to its highest *zT* value.

Taken together, the thermoelectric performance indicators demonstrate that the **PNTz4T** exhibits advantages in terms of electrical conductivity, while the **PNTzTT** demonstrates an optimized Seebeck coefficient and a lower thermal conductivity. These differences can be attributed to variations in phonon and charge transfer resulting from molecular stacking induced by backbone engineering. This comprehensive analysis highlights the potential of NTz‐based D‐A conjugated polymers for advanced thermoelectric applications, demonstrating significant progress in the backbone engineering of D‐A conjugated polymers.

## 3 Conclusion

This study elucidates the impact of backbone engineering on the thermoelectric properties of doped naphthobisthiadiazole (NTz)‐based donor–acceptor (D‐A) conjugated polymer thin films. The π‐conjugated system influences the optical properties and energy levels of three studied polymers (designated **PNTz4T**, **PNTzT**, and **PNTz3T**). The inclusion of thiophene and thieno[3,2‐b]thiophene units resulted in distinct red‐shift absorbance variations along with a reduced bandgap for the **PNTz4T** and **PNTzTT** compared to the **PNTz3T**. Molecular simulations revealed that the **PNTz3T** adopts a bent‐shaped arrangement, whereas the **PNTz4T** exhibits a linear arrangement. This results in different stacking modes, whereby the linear **PNTz4T** exhibits a predominantly edge‐on stacking due to ordered alignment of the side chains, while the bent **PNTz3T** molecules exhibit a face‐on orientation. The ordered alignment of side chains in the **PNTz4T** facilitates the dopant intercalation near the polymer backbone, which enhances the dopant interactions with the conjugated backbones. Meanwhile, the **PNTzTT** exhibits mixed edge‐on and face‐on orientations, which scatters the charge carriers and reduces their mobility, thereby resulting in the lowest thermal conductivity and the highest *zT* of 0.041. The doped **PNTz4T**, with its high carrier concentration due to the optimized dopant intercalation and its in‐plane mobility due to edge‐on stacking, exhibits a superior electrical conductivity (*σ*) and power factor (*PF*) of 88.3 S cm^−1^ and 34.2 µW m^−1^ K^−2^, respectively.

In brief, this study reveals numerous properties of pristine and doped NTz‐based D‐A conjugated copolymers, providing valuable insights for the design and synthesis of doped conjugated polymers for potential thermoelectric applications.

## 4 Experimental Section

### Synthesis of **PNTz3T**

To a reaction tube equipped with a stirring bar, 5,10‐bis[5‐bromo‐4‐(2‐decyltetradecyl)thiophen‐2‐yl]naphtho[1,2‐*c*:5,6‐*c'*]bis([1,2,5]thiadiazole) (**NTz2T‐Br_2_**) (62.0 mg, 0.05 mmol), 2,5‐bis(trimethylstannyl)thiophene (20.5 mg, 0.05 mmol), Pd(PPh_3_)_4_ (1.16 mg, 0.001 mmol) and toluene (2 mL) were added. The tube was purged with argon and sealed. The tube was then placed in a microwave reactor and heated at 200 °C for 2 h. After cooling to room temperature, the reaction mixture was poured into 50 mL of methanol containing 2 mL of hydrochloric and stirred for 1 h. The precipitate was then collected by filtration and subjected to sequential Soxhlet extraction with methanol, hexane, dichloromethane to remove the low molecular weight fractions. The residue was then extracted with chloroform. The extracted chloroform solution was concentrated and reprecipitated in methanol. Finally, the precipitate was collected by filtration and dried under vacuum to yield **PNTz3T** as a dark green solid (55 mg, 93%, *M*_n_ = 33200, *M*_w_ = 68900, *Đ* = 2.1).

### Synthesis of **PNTz4T**

The **PNTz4T** was synthesized in the same way as the **PNTz3T** by using **NTz2T‐Br_2_** and 5,5′‐bis(trimethylstannyl)‐2,2′‐bithiophene as the comonomers, yielding the polymer as a dark green solid (52 mg, 82%, *M*_n_ = 25600, *M*_w_ = 51000).

### Synthesis of **PNTzTT**

The **PNTzTT** was synthesized in the same way as the **PNTz3T** by using **NTz2T‐Br_2_** and 2,5‐bis(trimethylstannyl)thieno[3,2‐*b*]thiophene as the comonomers, yielding the polymer as a dark green solid (49 mg, 79%, *M*_n_ = 44500, *M*_w_ = 100000).

### Fabrication of Doped Conjugated Polymer Thin Films and TE Measurements

The polymer thin films were fabricated on 15 mm ×15 mm glass substrates. First, the glass substrates were cleaned by sequential sonication in deionized water, acetone, and isopropanol, followed by drying under a flow of nitrogen and oxygen plasma treatment for 10 min. Polymer solutions of **PNTz3T**, **PNTz4T**, and **PNTzTT** in chloroform (10 mg mL^−1^) were then spin‐coated at 2000 rpm for 15 s and annealed at 120 °C for 2 h. For the fabrication of the doped polymer thin films, the annealed films were each immersed in a solution of FeCl₃ in acetonitrile (3 mm) for 30 s and then dried under nitrogen to remove the residual solvent. The thermoelectric properties of the doped conjugated polymer thin films, including the Seebeck coefficient and electrical conductivity, were measured simultaneously using a commercial ZEM‐3 measurement system (ADVANCE RIKO Inc., Japan) at 323 K under a helium atmosphere. In the optimization process, the tests on doping time and concentration for polymers are conducted. The polymers showed similar thermoelectric performance at varying concentrations with the same doping time. However, significant differences were observed when the doping time was changed at a constant concentration. This led to identify optimal blending conditions for all three polymers, and the preliminary optimization results are summarized in Table S5 (Supporting Information). Additionally, the thermal conductivity of each sample was determined using a Linseis thin film laser flash analyzer (TF‐LFA) at room temperature.

## Conflict of Interest

The authors declare no conflict of interest.

## Supporting information



Supporting Information

## Data Availability

The data that support the findings of this study are available from the corresponding author upon reasonable request.
