# Supplementary material for: Controlling the Thermoelectric Performance of Doped Naphthobisthiadiazole‐Based Donor–Acceptor Conjugated Polymers through Backbone Engineering
Source: Adv Sci (Weinh). 2024 Nov 3;11(48):2410046. doi: 10.1002/advs.202410046 (PMC11672273; doi:10.1002/advs.202410046)
Supplement: Supplementary file 1 — Supporting Information [file ADVS-11-2410046-s001.docx]

Supporting Information

**Controlling Thermoelectric Performance of Doped Naphthobisthiadiazole-Based Donor-Acceptor Conjugated Polymers Through Backbone Engineering**

Jian-Fa Ding, Kodai Yamanaka, Shao-Huan Hong, Guan-Lin Chen, Wei-Ni Wu, Jhih-Min Lin, Shih-Huang Tung, Itaru Osaka,* and Cheng-Liang Liu*

J.-F. Ding, Dr. S.-H. Hong, W.-N. Wu, Prof. C.-L. Liu

Department of Materials Science and Engineering

National Taiwan University, Taipei, 10617, Taiwan

K. Yamanaka, Prof. I. Osaka

Graduate School of Advanced Science and Engineering

Hiroshima University, Higashi-Hiroshima, 739-8527, Japan

E-mail: iosaka@hiroshima-u.ac.jp

G.-L. Chen

Center for Condensed Matter Sciences

National Taiwan University, Taipei, 10617, Taiwan

Dr. J.-M. Lin

National Synchrotron Radiation Research Center

Hsinchu, 30076, Taiwan

Prof. S.-H. Tung

Institute of Polymer Science and Engineering

National Taiwan University, Taipei, 10617, Taiwan

Prof. C.-L. Liu

Advanced Research Center for Green Materials Science and Technology

National Taiwan University, Taipei, 10617, Taiwan

E-mail: liucl@ntu.edu.tw

**Materials:** All chemicals were used as purchased. **NTz2T-Br_2_**,^[1]^ 2,5-bis(trimethylstannyl)thiophene,^[2]^ 5,5′-bis(trimethylstannyl)-2,2′-bithiophene, 2,5-bis(trimethylstannyl)thieno[3,2-*b*]thiophene were synthesized according to the reported procedures.^[3-4]^ Molecular weights of the polymers were evaluated by a high-temperature GPC (180 ℃), TOSOH HLC-8321GPC/HT, using 1,2,4-trichlorobenzene (TCB) as the eluent and calibrated with polystyrene standard.

**Characterizations**: Nuclear magnetic resonance (NMR) spectra were taken on JNM-ECA500 (JEOL Ltd.), using C_6_D_4_Cl_2_ calibrated with the *o*-DCB peak at 6.94 ppm for ^1^H-NMR spectra. Thermogravimetric analysis (TGA) was performed with TGA/DSC 6200 (HITACHI). Differential scanning calorimetry (DSC) analysis was carried out with an Exstar DSC6200 thermal analyzer (Seiko Instruments) at a cooling and heating rate of 10 ℃ min^−1^. Ultraviolet-visible-near infrared (UV-Vis-NIR) spectra were recorded on a Hitachi U-4100 spectrophotometer. Cyclic voltammetry (CV) was carried out with an ALS Electrochemical Analyzer Model 612D. The CV measurements were performed in acetonitrile containing tetrabutylammonium hexafluorophosphate (Bu_4_NPF_6_, 0.1 M) as the supporting electrolyte. The counter and working electrodes were made of Pt, and the reference electrode was Ag/Ag^+^. The polymer thin films were directly formed on the working electrode. All potentials were calibrated with the standard ferrocene/ferrocenium redox couple (Fc/Fc^+^: *E*^1/2^ = +0.29 V measured under identical conditions). HOMO energy levels (*E*_HOMO_) and LUMO energy levels (*E*_LUMO_) were calculated with the following equations:

*E*_HOMO_ (eV) = −4.51 − *E*_ox_

*E*_LUMO_ (eV) = −4.51 + *E*_red_

where *E*_ox_ and *E*_red_ are the onset oxidation and reduction potentials of cyclic voltammograms, respectively. Ultraviolet photoelectron spectroscopy (UPS) and X-ray photoelectron spectra (XPS) measurements were conducted with a PHI 5000 VersaProbe III (ULVAC-PHI. Inc) under ultra-high vacuum 6.7×10^-8^ Pa. Excitation sources included the He I radiation (21.22 eV) and monochromatic Al Kα X-ray (1486.6 eV). AFM studies of pristine and doped polymer thin films were performed using Hitachi AFM5100N in tapping mode under ambient conditions, employing Hitachi SI-DF3PS tips with a resonant frequency of 70 kHz and a force constant of 2.0 N m^-1^. Grazing-incidence wide-angle X-ray scattering (GIWAXS) patterns were obtained at the TPS 25A beamlines at National Synchrotron Radiation Research Center (NSRRC, Taiwan). Thin film thicknesses were measured using an Alpha-Step® D-300 surface profiler (KLA-Tencor).

**Table S1.** A summary of Conductivity, Seebeck coefficient, and Power factor of previous p-type conjugated polymers doped with FeCl_3_ or F4TCNQ.

| Polymer | Dopant | Electrical ermal  (S cm^−1^) | Seebeck coefficient  (μV K⁻^1^) | Power factor  (μW m^-1^ K^-2^) | Reference |
| --- | --- | --- | --- | --- | --- |
| P3HT | FeCl_3_ | 105 ± 12 | 42 ± 3 | 46 ± 7 | [5] |
| PDPP3T |  | 217 ± 8 | 52 ± 3 | 247 ± 21 |  |
| pBBT-2T-2T |  | 313.9 | 32.8 | 43.5 | [6] |
| pBBT-2T-TT |  | 232.7 ± 23.7 | 49.4± 3.6 | 56.5 ± 4.3 |  |
| PDPP-3T |  | 0.28 ± 0.04 | 242 ± 9 | 1.6 ± 0.1 | [7] |
| PDPP-4T-EDOT |  | 271.5 ± 7.4 | 174.2 ± 16.6 | 298.2 ± 40.2 | [8] |
| PDPP-5T |  | 119.6 ± 6.1 | 42.2 ± 0.6 | 11.1 ± 0.6 |  |
| PC16BTF |  | 72.1 | 59.9 | 17.5 | [9] |
| PC12BTH |  | 76.3 | 49.7 | 18.8 |  |
| PC16BTH |  | 118.7 | 43.5 | 22.4 |  |
| PC20BTH |  | 98.4 | 46.5 | 21.2 |  |
| P(BDTTT-DPP) |  | 1.83 ± 0.55 | 193.66 ± 53.60 | 6.50 ± 1.97 | [10] |
| P(BDT-DPP) |  | 0.1 ± 0.09 | 160.95 ± 2.45 | 0.26 ± 0.08 |  |
| pBBTa26-2T | F4TCNQ | 43.1 | 79.2 | 11.8 | [11] |
| pBBTa26-4T |  | 14.1 | 31.5 | 1.2 |  |
| pBBTa26-TT |  | 102.1 | 57.0 | 6.8 |  |
| P3HT |  | 0.61 | 105 | 0.76 | [12] |
| PCDTPT |  | 5.13 | 194 | 21.8 |  |
| PCDTFBT |  | 8.73 | 181 | 31.5 |  |

Table S2. Summary of thermoelectric performance for doped polymer thin films.

| Sample | *σ*  (S cm^-1^) | *S*  (μV K^-1^) | *PF*  (μW m^-1^ K^-2^) | *κ*  (W m^-1^ K^-1^) | *zT* |
| --- | --- | --- | --- | --- | --- |
| PNTz3T | 57.7 | 62.5 | 22.5 | 0.28 | 0.023 |
| PNTz4T | 88.3 | 62.2 | 34.2 | 0.31 | 0.032 |
| PNTzTT | 53.2 | 70.6 | 26.5 | 0.19 | 0.041 |

Table S3. Thermal conductivity of pristine and doped conjugated polymer thin films.

| *κ*  (W m^-1^ K^-1^) | PNTzTT | PNTz4T | PNTz3T |
| --- | --- | --- | --- |
| Pristine | 0.169 | 0.287 | 0.243 |
| Doped | 0.192 | 0.313 | 0.286 |

**Table S4.** Hall effect measurement of doped conjugated polymer thin films.

| Sample | *μ*  (cm^2^ V^-1^ s^-1^) | *n*  (cm^-3^) | *σ*  (S cm^-1^) |
| --- | --- | --- | --- |
| PNTz3T | 2.7 × 10^-1^ | 4.2 × 10^20^ | 18.4 |
| PNTz4T | 3.2 × 10^-1^ | 5.2 × 10^20^ | 26.9 |
| PNTzTT | 2.4 × 10^-1^ | 4.0 × 10^20^ | 15.1 |

**Table S5.** Results of various doping concentrations for doped polymers.

|  | Thickness (Å) | σ (S cm^-1^) | S (μV K^-1^) | PF (μW m^-1^ K^-2^) |
| --- | --- | --- | --- | --- |
| PNTz3T (15 s) | 1024.8 | 16261.7 | 104.21 | 3.48 |
| PNTz3T (30 s) | 1097.1 | 8211.895 | 107.64 | 6.86 |
| PNTz4T (15 s) | 906.9 | 3383.215 | 64.88 | 7.32 |
| PNTz4T (30 s) | 982.3 | 2186.785 | 74.73 | 13.87 |
| PNTzTT (15 s) | 924.3 | 18854.4 | 121.30 | 4.50 |
| PNTzTT (30 s) | 894.2 | 19649.09 | 131.38 | 5.24 |

**Table S6.** The element composition of doped PNTz3T.

| Element | Peak (eV) | FWHM (eV) | Atomic (%) |
| --- | --- | --- | --- |
| C1s | 284.38 | 1.18 | 78.54 |
| C1s Scan A | 285.6 | 1.06 | 5.59 |
| C1s Scan B | 283.4 | 1.21 | 3.26 |
| Cl2p | 198.32 | 1.39 | 0.57 |
| Cl2p Scan A | 201.49 | 1.28 | 0.09 |
| Cl2p Scan B | 199.86 | 1.58 | 0.57 |
| Fe2p3 | 710.71 | 2.98 | 0.99 |
| Fe2p3 Scan A | 714.55 | 3.37 | 0.5 |
| Fe2p3 Scan B | 709.23 | 1.78 | 0.39 |
| N1s | 399.06 | 1.19 | 3.53 |
| N1s Scan A | 400.4 | 0.91 | 0.18 |

**Table S7.** The element composition of doped PNTz4T.

| Element | Peak (eV) | FWHM (eV) | Atomic (%) |
| --- | --- | --- | --- |
| C1s | 284.13 | 1.09 | 79.8 |
| C1s Scan A | 285.3 | 0.93 | 6.05 |
| C1s Scan B | 283.2 | 1.14 | 3.24 |
| Cl2p | 199.31 | 2.24 | 0.1 |
| Cl2p Scan A | 197.92 | 1.04 | 0.03 |
| Fe2p3 | 709.28 | 2.28 | 0.1 |
| Fe2p3 Scan A | 714.81 | 3.37 | 0.07 |
| Fe2p3 Scan B | 711.29 | 3.37 | 0.09 |
| N1s | 398.76 | 1 | 3.56 |
| N1s Scan A | 399.48 | 2.07 | 0.89 |
| N1s Scan B | 397.75 | 1.28 | 0.28 |

**Table S8.** The element composition of doped PNTzTT.

| Element | Peak (eV) | FWHM (eV) | Atomic (%) |
| --- | --- | --- | --- |
| C1s | 284.14 | 1.08 | 79.69 |
| C1s Scan A | 285.3 | 0.94 | 6.43 |
| C1s Scan B | 283.2 | 1.14 | 3.12 |
| Cl2p | 199.16 | 2.48 | 0.13 |
| Cl2p Scan A | 197.95 | 0.98 | 0.02 |
| Fe2p3 | 712.49 | 3.37 | 0.06 |
| Fe2p3 Scan A | 715.82 | 3.37 | 0.07 |
| Fe2p3 Scan B | 709.61 | 2.65 | 0.11 |
| N1s | 398.78 | 0.99 | 3.49 |
| N1s Scan A | 399.5 | 1.86 | 0.76 |
| N1s Scan B | 397.79 | 1.26 | 0.35 |

N

S

N

N

S

N

S

S

C

10

H

21

C

12

H

25

C

10

H

21

C

12

H

25

Br

Br

N

S

N

N

S

N

S

S

C

10

H

21

C

12

H

25

C

10

H

21

C

12

H

25

S

*n*

N

S

N

N

S

N

S

S

C

10

H

21

C

12

H

25

C

10

H

21

C

12

H

25

S

S

*n*

N

S

N

N

S

N

S

S

C

10

H

21

C

12

H

25

C

10

H

21

C

12

H

25

S

S

*n*

Pd

(PPh

3

)

4

T

oluene

200 ¢XC, 2 h

(Mi

cro

w

a

ve

)

S

S

Sn

Me

3

Me

3

Sn

S

S

Sn

Me

3

Me

3

Sn

S

Sn

Me

3

Me

3

Sn

Pd

(PPh

3

)

4

T

oluene

200 ¢XC, 2 h

(Mi

cro

w

a

ve

)

Pd

(PPh

3

)

4

T

oluene

200 ¢XC, 2 h

(Mi

cro

w

a

ve

)

P

N

T

z

3

T

P

N

T

z

4

T

P

N

T

z

T

T

N

T

z

2

T

-

B

r

2

Scheme S1. Synthetic route to the polymers.

Figure S1. The molecule structures of NTz-based conjugated polymers for organic solar cells (OSCs) and organic field-effect transistors (OFETs).

Figure S2. ^1^H NMR spectra of the polymers.

**Figure S3.** (a) TGA curves and (b) DSC curves of the polymers.

Figure S4. Solution-state UV-vis-NIR absorption spectra of pristine PNTz3T, PNTz4T and, PNTzTT

Figure S5. Cyclic voltammograms of PNTz3T, PNTz4T and, PNTzTT. *E*_ox_ and *E*_red_ were 0.61 V and −1.63 V for PNTz3T, 0.59 V and −1.68 V for PNTz4T, and 0.55 V and −1.60 V for PNTzTT. *E*_HOMO_ and *E*_LUMO_ calculated by using the following equations: *E*_HOMO_ = −4.80 − *E*_ox_, *E*_LUMO_ = −4.80 − *E*_red_.

**
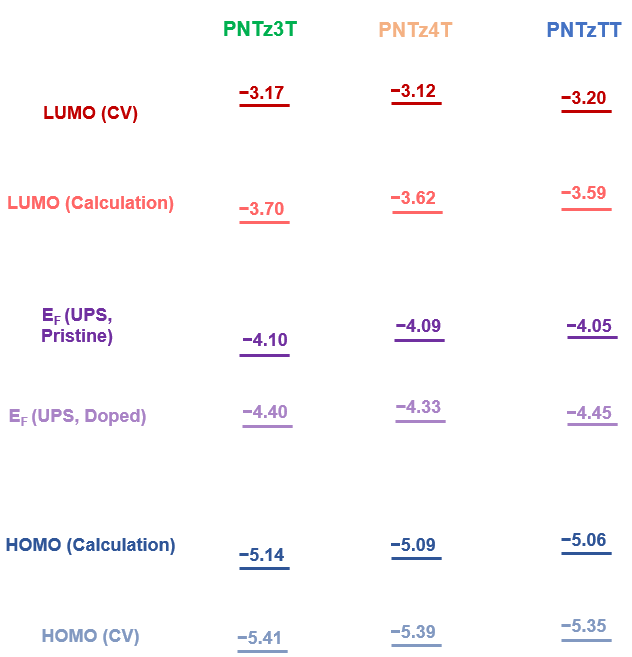
**

**Figure S6.** Energy level diagrams for samples before and after doping.

**
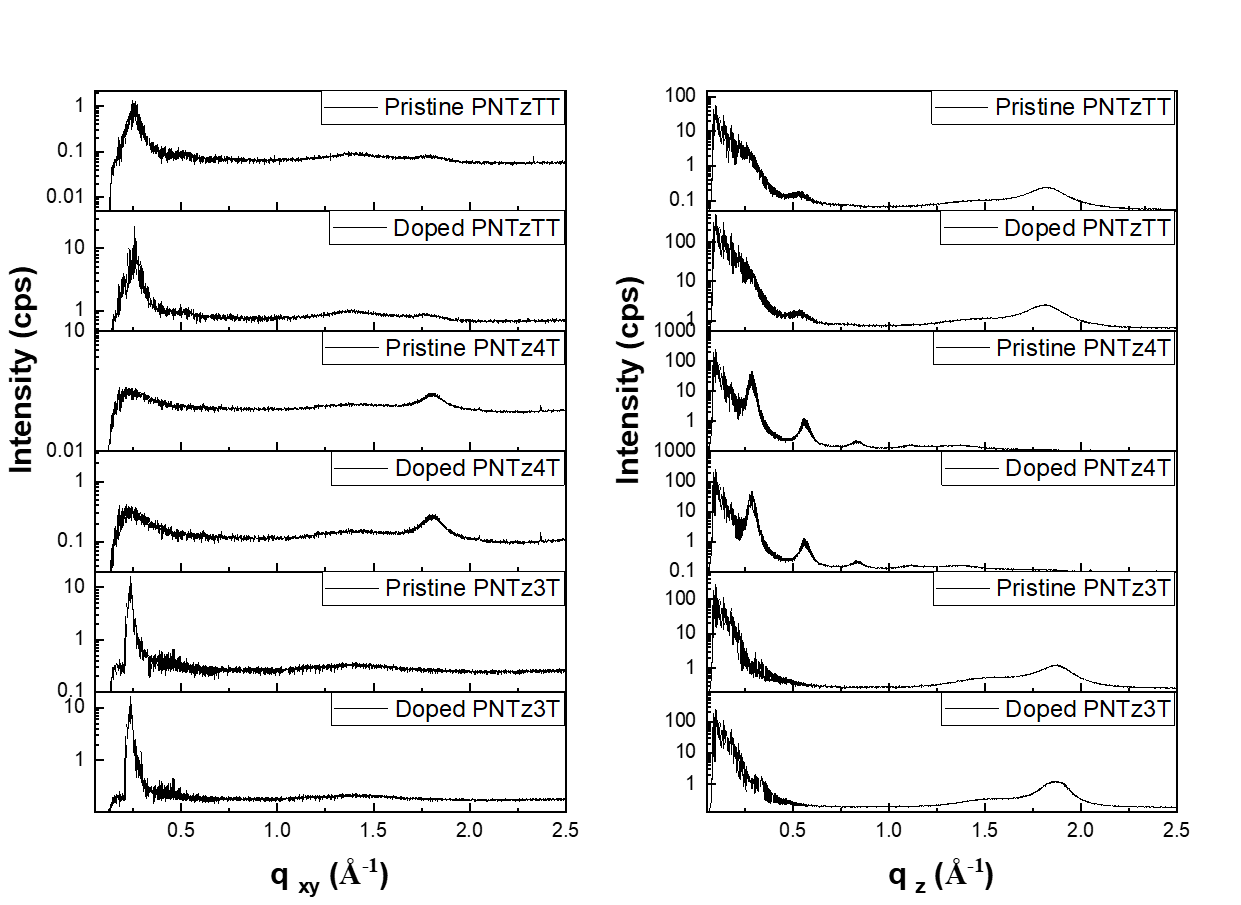
**

**Figure S7.** GIWAXS in-plane and out-of-plane line cut pattern of pristine and doped polymer thin films.

**Figure S8.** Pole figures of the (100) peak for films spun on PNTz3T, PNTZ4T, and PNTzTT.

References

[1] I. Osaka, M. Shimawaki, H. Mori, I. Doi, E. Miyazaki, T. Koganezawa, K. Takimiya, *J. Am. Chem. Soc.* **2012**, *134*, 3498.

[2] K. Foo, E. Sella, I. Thomé, M. D. Eastgate, P. S. Baran, *J. Am. Chem. Soc.* **2014**, *136*, 5279.

[3] X. Hu, M. Shi, J. Chen, L. Zuo, L. Fu, Y. Liu, H. Chen, *Macromol. Rapid Commun.* **2011**, *32*, 506.

[4] M. Sato, A. Asami, G. Maruyama, M. Kosuge, J. Nakayama, S. Kumakura, T. Fujihara, K. Unoura *J. Organomet. Chem.* **2002**, 56, 654.

[5] I. H. Jung, C. T. Hong, U. H. Lee, Y. H. Kang, K. S. Jang, S. Y. Cho, *Sci. Rep.* **2017**, *7*, 44704.

[6] T. L. D. Tam, G. Wu, S. W. Chien, S. F. V. Lim, S.-W. Yang, J. Xu, *ACS Mater. Lett.* **2020**, *2*, 147.

[7] H. Zeng, M. Mohammed, V. Untilova, O. Boyron, N. Berton, P. Limelette, B. Schmaltz, M. Brinkmann, *Adv. Electron. Mater.* **2021**, *7*.

[8] Z. Liu, Y. Hu, P. Li, J. Wen, J. He, X. Gao, *J. Mater. Chem. C* **2020**, *8*, 10859.

[9] J.-F. Ding, G.-L. Chen, P.-H. Liu, K.-W. Tseng, W.-N. Wu, J.-M. Lin, S.-H. Tung, L. Wang, C.-L. Liu, *J. Mater. Chem. A* **2024**, *12*, 9806.

[10] B. Li, X. Li, F. Yang, Y. Chen, X. Mao, S. Wan, H. Xin, S. Yan, M. Wang, C. Gao, L. Wang, *ACS Appl. Energy Mater.* **2021**, *4*, 4662.

[11] T. L. D. Tam, C. K. Ng, S. L. Lim, E. Yildirim, J. Ko, W. L. Leong, S.-W. Yang, J. Xu, *Chem. Mater.* **2019**, *31*, 8543.

[12] E. H. Suh, Y. J. Jeong, J. G. Oh, K. Lee, J. Jung, Y. S. Kang, J. Jang, *Nano Energy* **2019**, *58*, 585.
